# Investigation of myositis and scleroderma specific autoantibodies in patients with lung cancer

**DOI:** 10.1186/s13075-018-1678-9

**Published:** 2018-08-09

**Authors:** Zoe E. Betteridge, Lynsey Priest, Robert G. Cooper, Neil J. McHugh, Fiona Blackhall, Janine A. Lamb

**Affiliations:** 1 Department of Pharmacy and Pharmacology, University of Bath, Bath, UK; 2 Division of Molecular and Clinical Cancer Sciences, University of Manchester, Manchester, UK; 3 MRC/ARUK Centre for Integrated Research into Musculoskeletal Ageing, University of Liverpool, Liverpool, UK; 4 Royal National Hospital for Rheumatic Diseases, Royal United Hospitals Foundation Trust, Bath, UK; 5 CRUK Lung Cancer Centre of Excellence, The Christie NHS Foundation Trust, Wilmslow Road, Manchester, UK; 6 Centre for Epidemiology, Faculty of Biology, Medicine and Health, Manchester Academic Health Science Centre, University of Manchester, Manchester, UK

**Keywords:** Idiopathic inflammatory myopathies, Myositis, Scleroderma, Cancer, Autoantibodies, Anti-glycyl-tRNA synthetase

## Abstract

**Background:**

The close temporal association between onset of some connective tissue diseases and cancer suggests a paraneoplastic association. Adult patients with scleroderma with anti-RNA polymerase III autoantibodies and adult patients with dermatomyositis with anti-transcriptional intermediary factor 1 (anti-TIF1) or anti-nuclear matrix protein 2 (anti-NXP2) autoantibodies have a significantly increased risk of developing cancer. Autoantibodies may serve as biomarkers for early detection of cancer and also could be relevant for prediction of responses to immune therapies. We aimed to test whether myositis and scleroderma specific or associated autoantibodies are detectable in individuals with lung cancer.

**Methods:**

Serum from 60 Caucasian patients with lung cancer (30 with small cell lung cancer, 30 with non-small cell lung cancer) was screened for myositis and scleroderma specific and associated autoantibodies by radiolabelled immunoprecipitation.

**Results:**

Anti-TIF1, anti-NXP2 or anti-RNA polymerase III autoantibodies were not detected in any of the 60 patients with lung cancer. Anti-glycyl-transfer RNA (tRNA) synthetase (anti-EJ) autoantibodies were detected in one patient with non-small cell lung cancer. No other known myositis or scleroderma autoantibodies were identified.

**Conclusions:**

Myositis and scleroderma specific autoantibodies, including anti-TIF1, anti-NXP2 and anti-RNA polymerase III, are rare in patients with lung cancer without an autoimmune disease. We report here the first case of anti-EJ autoantibodies being detected in a patient with lung cancer without clinical or radiographic evidence of the anti-synthetase syndrome.

**Electronic supplementary material:**

The online version of this article (10.1186/s13075-018-1678-9) contains supplementary material, which is available to authorized users.

## Background

A close temporal association has been observed between the onset of some autoimmune connective tissue diseases (CTD) and various cancers, suggesting that the appearance of a CTD may sometimes represent a paraneoplastic phenomenon. For example, adult patients with dermatomyositis (DM) with anti-transcription intermediary factor 1 (anti-TIF1) autoantibodies have a dramatically increased risk of developing cancer compared to the general population [1]. Presence of anti-nuclear matrix protein 2 (anti-NXP2) autoantibodies similarly is associated with an increased cancer risk in adult DM [2]. A meta-analysis of five studies [3] showed that the pooled standardized incidence ratio for lung cancer among patients with DM is 19.74 (95% confidence interval 18.91–20.58), second only to lymphatic and haematopoietic cancers.

DM comprises a subgroup of the idiopathic inflammatory myopathies (IIM), a rare CTD spectrum characterized by inflammation of skeletal muscle (myositis) causing weakness and associated disability. Extra-muscular manifestations occur in the lungs and skin, and temporally associated cancers are common. In addition to clinical classification, IIM can be accurately classified according to the presence of approximately 20 myositis-specific and associated autoantibodies (MSA/MAA), identified in 60–70% of adult patients with IIM. MSA are almost mutually exclusive, and predictive of an individual patient’s clinical phenotype, including disease progression and treatment response characteristics [4]. “Cancer associated myositis” has been defined as myositis occurring in association with an incident cancer diagnosed 3 years either side of myositis onset, and a treatment-induced cancer cure can be associated with a simultaneous regression of the myositis. Recent evidence suggests that scleroderma also represents a paraneoplastic phenomenon, being similarly temporally associated with cancers in some anti-RNA polymerase III-positive patients [5]. The mechanism underlying this close temporal association between cancer onset and certain serologically defined subgroups of myositis and scleroderma is unknown.

In patients with cancer, a large number of autoantibodies directed against intracellular tumour-associated antigens have been identified. These autoantibodies may be detected in patients’ serum prior to the initial presentation of clinical cancer signs. Current theories suggest that autoantibody production in patients with cancer may result from changes in tissue protein expression, altered protein structure, defects in tolerance and inflammation and aberrant tissue degradation mechanisms [6]. Inflammation in the tumour microenvironment may facilitate the release of certain intracellular antigens to create reactive neo-epitopes via aberrant protein expression and/or altered protein structure through somatic mutation or conformational change. These changes facilitate the generation of autoantibodies against these altered proteins.

For paraneoplastic rheumatic diseases, a model has been proposed whereby a mutation in an incident cancer causes an increase in neo-antigen expression, triggering an autoimmune anti-tumour cytolytic response. This leads to successful autoimmune-mediated elimination of the responsible cancer in some patients [7]. Although the malignancy may be the antigen source initiating the autoimmune anti-tumour cytolytic response, a cross-reaction against regenerating cells in other target tissues (e.g. muscle and skin cells in patients with dermatomyositis) may initiate a self-propagating “feed-forward” loop of tissue damage and induced autoimmunity in genetically susceptible individuals. These observations support the “immunosurveillance” hypothesis; the continual eradication of nascent tumour cells via immunoediting and triggering of cellular and humoral immune responses.

In view of the possible association between incident cancers and induced secondary autoimmunity, we undertook a pilot study to ascertain whether serotyping by the gold standard of radiolabelled immunoprecipitation (IP) would uncover evidence of clinically covert CTD-associated autoantibody generation in patients with primary lung cancer and without clinically overt connective tissue disease.

## Methods

### Patient cohort

Caucasian patients with lung cancer had already been recruited via the ChemoRes EU Framework 6 integrated project (https://www.cruk.manchester.ac.uk/Our-Research/CEP/Areas-of-Interaction-with-Other-Teams), designed to improve the outcome of cancer chemotherapy. These patients were recruited into a prospective, single-centre study being conducted at the Christie Hospital, Manchester, UK. To be eligible for ChemoRes study inclusion, small cell lung cancer (SCLC) had to be histologically or cytopathologically confirmed, and disease had to naïve to chemotherapy and staged and managed using standard treatment protocols. Non-small cell lung cancer (NSCLC) had to histologically proven and radiologically confirmed as stage IIIA, IIIB or IV disease, and also had to be naïve to chemotherapy [8]. For our pilot serological study we opportunistically recruited 30 each of these patients with SCLC and NSCLC from ChemoRes. All patients gave their written, informed consent to the ethically approved study protocols. Peripheral blood samples for IP testing were collected in the 7 days prior to commencing their planned ChemoRes treatment, and serum was separated according to standard operating procedures and good clinical laboratory practice. Clinical data including age, gender, ethnicity, diagnosis and smoking status were collected.

### Autoantibody testing

Autoantibody testing for myositis and scleroderma specific and associated autoantibodies: Jo-1, PMScl, snRNP, Mi-2, Ku, PL12, PL7, EJ, KS, OJ, Zo, Ha, Topo, U3, SRP, TIF1, SAE, MDA5, Ro60, La, RNA Polymerase I-III, AMA, Th/To, NXP2, EIF3 and EIF2B, was carried out by IP using radiolabelled ^35^S-methionine on patient serum, as described previously [9]. Serum from patients positive for known myositis and scleroderma autoantibodies were included as controls. Antibody positivity by IP was confirmed using the EUROLINE Autoimmune Inflammatory myopathies (IgG) recombinant line-blot technology (Euroimmun, Lübeck, Germany).

When patients immunoprecipitated bands at 140 kDa/155 kDa, their samples were screened also by TIF1γ ELISA using rTIF1γ (Origene, USA), according to standard protocols. All samples were tested in duplicate with a positive cutoff defined as > 3 SD above the mean of 40 healthy controls.

## Results

Demographic data for the two patient cohorts are shown in Table 1. Myositis specific autoantibodies against glycyl-transfer RNA (tRNA) synthetase (anti-EJ) were identified by IP in one patient with NSCLC (Additional file 1: Figure S1). This antibody specificity was also positive by line-blot testing, with a semi-quantitative value of +++. Although TIF1γ autoantibodies have been strongly associated with malignancy in dermatomyositis, none of the 60 patients with lung cancer tested positive for anti-TIF1, anti-NXP2 or anti-RNAP III autoantibodies (by either IP or TIF1γ ELISA), indicating that these autoantibodies are rare in patients with lung cancer without an autoimmune disease. No other known myositis or scleroderma autoantibodies were identified.

**Table 1:** Patient demographics

Characteristic	SCLC (*n* = 30)	NSCLC (*n* = 30)
Age at onset, years		
Median	68	67
Range	48–82	53–78
Sex		
Female	18	15
Male	12	15
Diagnosis		
Limited	13	
Extensive	17	
Adenocarcinoma		12
Squamous cell carcinoma		8
Other		2
Not documented		8
Smoking status		
Current smoker	14	6
Former smoker	14	14
Never-smoker	0	3
Not documented	2	7

*SCLC* small cell lung cancer, *NSCLC* non-small cell lung cancer

A total of 55 patients (including the patient with anti-EJ) had one or more unknown bands on IP, which did not correspond to any known CTD autoantigen. Although these were generally moderate or strong bands, indicating the possible presence of an autoantibody, 10 patients (5 with SCLC and 5 with NSCLC) had only weak bands thought to represent non-specific binding: the remaining 5 patients were completely negative by IP; all had SCLC.

### Anti-glycl-tRNA synthetase (anti-EJ)-positive patient

The anti-EJ-positive patient was female, and aged 67 years. She was a former smoker, starting at age 17 years, but stopped 2 years prior to her lung cancer diagnosis. The patient originally presented with right arm and shoulder discomfort with reduced shoulder movements, initially diagnosed as arthritis. She had normal exercise tolerance at presentation. A computerized tomography (CT) scan revealed a large spiculated mass in the posterior segment of the right upper lobe. Significantly enlarged lymph nodes were seen in the supraclavicular, right paratracheal, prevascular, subcarinal and right hilar regions. The CT scan showed no evidence of interstitial lung disease. Histological analysis confirmed poorly differentiated adenocarcinoma. Immunohistochemical analysis demonstrated positivity for cytokeratin 7, thyroid transcription factor-1 and carcinoembryonic antigen 125. Tumour cells were negative for cytokeratin 20, gross cystic disease fluid protein 15 and oestrogen receptor, in keeping with metastatic adenocarcinoma, primary non-small cell lung cancer. Somatic mutation in the epidermal growth factor receptor (*EGFR*) gene or presence of anaplastic lymphoma receptor tyrosine kinase (*ALK*) gene fusion was not present. Following four cycles of carboplatin and gemcitabine, follow-up CT demonstrated progressive disease with increase in the size of the lung mass plus satellite lesions plus a new right pleural effusion, even though the patient had improved symptomatically. The patient survived 155 days (5.1 months) from the date of her consent to participate in the ChemoRes study.

## Discussion

In this pilot study, we used IP to interrogate for the presence of myositis and scleroderma specific and associated autoantibodies in individuals with primary lung cancer. We identified one patient with NSCLC with autoantibodies against glycyl-tRNA synthetase (anti-EJ), subsequently confirmed by line-blot testing. No other CTD autoantibodies were identified, including antibodies against TIF1γ, NXP2 or RNAP III. We conclude that known myositis and scleroderma specific and associated autoantibodies are rare in patients with lung cancer without a known CTD. These results are in keeping with those from a recent study that similarly tested for anti-TIF1γ, anti-NXP2, and anti-RNAP III autoantibodies in patients with breast cancer without rheumatic disease [10]. Taken together, these findings suggest that immune-mediated anti-tumor cytolytic responses in patients with cancer rarely induce paraneoplastic CTDs, or that patients with cancer without an associated CTD fail to mount an immune response sufficiently strong to induce paraneoplastic consequences. Although five patients in our study were autoantibody negative by IP, the remaining patients had unidentified bands, potentially indicating the presence of autoantibodies targeting unknown antigens. This is consistent with the large number of autoantibodies that have been reported in cancer, and the role of immune homeostasis and a humoral immune response in cancer pathogenesis [6].

To our knowledge, this is the first report of anti-glycyl-tRNA synthetase autoantibodies in a patient without a known anti-synthetase syndrome. Anti-EJ autoantibodies are identified in only ~ 1% of adult Caucasian patients with IIM [4].

A limitation of this pilot study is the lack of follow-up clinical data relating to complete ascertainment of CTD or anti-synthetase syndrome development; the patient seropositive for anti-EJ lived only 155 days following consent to participate in the ChemoRes study. Patients were recruited as part of a project to improve the outcome of cancer chemotherapy, and so were referred to oncologists rather than rheumatologists. Subtle CTD clinical features therefore could have been overlooked. Alternatively, anti-EJ autoantibodies may have been detectable in this patient’s serum prior to the development of overt CTD clinical signs, as reported in patients seropositive for rheumatoid factor before they develop overt rheumatoid arthritis. The median age of lung cancer onset in this pilot study was 68 years and 67 years in SCLC and NSCLC, respectively. In a large international study of IIM, the mean age of IIM onset was ~ 50 years, and was 57 years in those with malignancy, with a median interval between IIM onset and associated cancer diagnoses of only 1 month [11], suggesting possible selection against patients with cancer-associated myositis in the present study.

There is currently an unmet clinical need for biomarkers to facilitate early detection of lung and other cancers. Recent progress in the treatment of lung cancer with immune therapies reinforces the need to better understand host immune responses in the context of lung cancer. Although the presence of a cancer-associated autoantibody or paraneoplastic syndrome could potentially signify a tumuor more likely to respond to an immunomodulatory approach, the low prevalence of CTD autoantibodies identified in this series counters their potential utility in an unselected population. Notably, however, the relatively small number of patients with cancer included in this study limits our ability to detect rare or low-frequency autoantibodies; utility in screening should be addressed using larger cohorts with longitudinal follow-up data.

## Conclusions

In patients with lung cancer without an associated CTD, myositis and scleroderma specific and associated autoantibodies, including anti-TIF1, anti-NXP2 and anti-RNAP III, are rare. We identified one patient with NSCLC with anti-EJ autoantibodies, the first time that this anti-synthetase autoantibody has been reported in a patient without anti-synthetase syndrome clinical features.

## Additional file

Additional file 1: **Figure S1.** Radio-immunoprecipitation of NSCLC samples and positive controls. A Autoradiograph of a 10% SDS-PAGE, loaded with immunoprecipitates using either serum containing known autoantibodies (Lane 1: Healthy Control/Normal Serum (NS), Lane 2: anti-Jo-1 and anti-U1RNP/Sm, Lane 3: anti-PMScl, anti-Ro60 and anti-La, Lane 4: anti-Mitrochondrial autoantibodies (AMAs), Lane 5; anti-Ku and anti-Mi-2), or NSCLC samples screened as part of this study (lanes 6–14). The sample loaded into lane 11 (NSCLC269, marker with *) contains anti-EJ autoantibodies. B Autoradiograph of a 10% SDS-PAGE, loaded with immunoprecipitates using serum known to contain anti-EJ autoantibodies (lanes 1–4) or NSCLC269 identified as containing anti-EJ. (PPTX 309 kb)

## Abbreviations

CT: Computerized tomography
CTD: Connective tissue disease
DM: Dermatomyositis
ELISA: Enzyme-linked immunosorbent assay
IIM: Idiopathic inflammatory myopathies
IP: Immunoprecipitation
MSA/MAA: Myositis specific/myositis associated autoantibodies
NSCLC: Non-small cell lung cancer
NXP2: Nuclear matrix protein 2
SCLC: Small cell lung cancer
TIF1: Transcriptional intermediary factor 1
tRNA: Transfer RNA
