# Supplementary figures and images for: Investigation of myositis and scleroderma specific autoantibodies in patients with lung cancer
Source: Arthritis Res Ther. 2018 Aug 9;20:176. doi: 10.1186/s13075-018-1678-9 (PMC6085683; doi:10.1186/s13075-018-1678-9)

## Slide 1
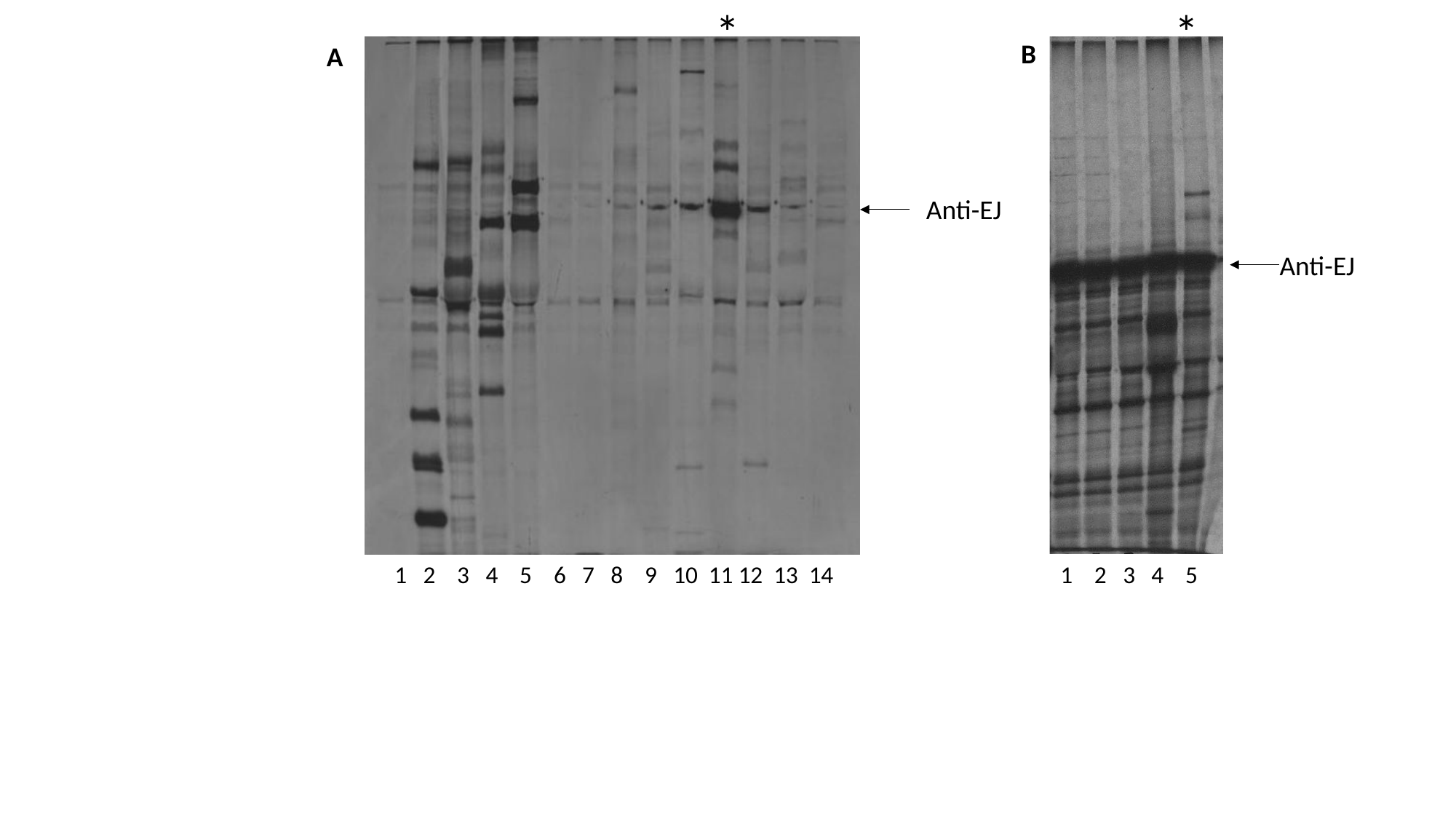

*
*
B
A
Anti-EJ
Anti-EJ
1 2 3 4 5 6 7 8 9 10 11 12 13 14
1 2 3 4 5

Supplement: Supplementary file 1 — Figure S1. Radio-immunoprecipitation of NSCLC samples and positive controls. A Autoradiograph of a 10% SDS-PAGE, loaded with immunoprecipitates using either serum containing known autoantibodies (Lane 1: Healthy Control/Normal Serum (NS), Lane 2: anti-Jo-1 and anti-U1RNP/Sm, Lane 3: anti-PMScl, anti-Ro60 and anti-La, Lane 4: anti-Mitrochondrial autoantibodies (AMAs), Lane 5; anti-Ku and anti-Mi-2), or NSCLC samples screened as part of this study (lanes 6–14). The sample loaded into lane 11 (NSCLC269, marker with *) contains anti-EJ autoantibodies. B Autoradiograph of a 10% SDS-PAGE, loaded with immunoprecipitates using serum known to contain anti-EJ autoantibodies (lanes 1–4) or NSCLC269 identified as containing anti-EJ. (PPTX 309 kb) [file 13075_2018_1678_MOESM1_ESM.pptx]
